# In-Plane Strengthening of Unreinforced Masonry Walls with Discrete Glass Fiber-Reinforced Polymer Grid Strips Bonded with Sprayed Polyurea

**DOI:** 10.3390/ma18040771

**Published:** 2025-02-10

**Authors:** Piyong Yu, Pedro Silva, Antonio Nanni

**Affiliations:** 1 College of Architecture and Civil Engineering, Beijing University of Technology, Beijing 100124, China; 2 Department of Civil and Environmental Engineering, George Washington University, Washington, DC 20052, USA; silvap@gwu.edu; 3 Department of Civil and Architectural Engineering, University of Miami, Coral Gables, FL 33146, USA; nanni@miami.edu

**Keywords:** GFRP grid, strips, polyurea, compression, strengthening, URM walls

## Abstract

In this study, unreinforced masonry (URM) walls constructed from concrete blocks and clay bricks were strengthened using horizontally and vertically oriented glass fiber-reinforced polymer (GFRP) grid strips bonded with sprayed polyurea. The walls were subjected to diagonal compression loading until failure. The results demonstrated a significant improvement in both the shear capacity and pseudo-ductility of the strengthened URM walls compared to their unstrengthened counterparts. The primary conclusions drawn from this research are as follows: (1) the maximum strain in the vertical GFRP strips increased with the higher axial stiffness of the strips; (2) the discrete vertical strips contributed substantially to enhancing the shear capacity and pseudo-ductility of the URM walls; (3) increasing the axial stiffness of the vertical strips can alter the failure mode of the walls, shifting it from joint failure to tension or compression failure of the blocks or bricks; (4) a reduction factor is necessary to account for the potential asymmetrical performance of double-sided strengthening schemes applied to URM walls. The experimental program was reported in a previous publication and additional information is presented in this paper.

## 1. Introduction

Large inventories of older masonry buildings worldwide have been designed without proper seismic design standards. Previous research and seismic events around the world have clearly demonstrated that, if left in its current state, unreinforced masonry (URM) walls are prone to extensive damage during seismic events. Consequently, the seismic upgrade of older URM walls has become a critical issue in contemporary structural engineering. Fiber-reinforced polymer (FRP) composites have gained widespread acceptance as a solution for the seismic strengthening of URM walls [1,2,3]. However, limitations in displacement ductility capacity and other drawbacks of FRP materials have prompted the investigation of alternative strengthening systems, such as fabric-reinforced cementitious matrix (FRCM), also known by various other names including textile-reinforced mortar (TRM), cementitious matrix-grid (CMG), or composites reinforced mortar [4,5,6,7,8,9], steel-reinforced grout (SRG), and engineered cementitious composites (ECC) [10,11].

Research on the strengthening of URM walls with polyurea has been reported recently.

Polyurea combines good application properties, such as rapid cure and insensitivity to substrate moisture, with equally good physical properties, such as high hardness, flexibility, tensile strength, and resistance against cracks [12]. Hrynyk and Myers [13] presented that sprayed polyurea was effective in improving the energy absorption and reducing the fragmentation of URM walls. Cuong et al. [14] found that polyurea clearly increased the shear strength of masonry prisms. Zhu et al. [15] studied clay brick walls strengthened with sprayed polyurea under close-in blast load and found that polyurea was effective in increasing the load capacity. However, investigation of the sprayed polyurea in the shear strengthening of URM walls is extremely limited in the open literature.

Regarding the shear capacity of URM walls, it is generally accepted that the contribution of vertical fiber-reinforced polymer (FRP) strips to the shear strength is negligible. Triantafillou [16] concluded that the strengthening of URM walls with discrete vertical narrow FRP strips or bars was ineffective, as the dowel action provided by the FRP system was minimal and did not significantly contribute to the shear strength of the walls. Similarly, Prota et al. [17] and D’Ambra et al. [18] reported that, when vertical strips or fibers have no continuity at the ends, the contribution of vertical FRP can be neglected in the shear strength of URM walls. By contrast, Borri et al. [10] found a significant increase in the shear strength of clay brick walls after being strengthened with discrete steel grout strips using both horizontal and vertical layouts. However, their analysis only considered the contribution of the horizontal reinforcement in calculating the shear strength.

Del Zoppo et al. [11] reported that horizontal fibers mainly affect the shear strength and stiffness of panels; whereas vertical fibers mainly contribute to the deformation capacity of URM walls in the post-peak phase. Meriggi et al. [19] concluded that vertical strips are effective in redistributing stress in FRCM and can prevent shear sliding by preventing vertical separation at mortar joints and uplift that are associated with the horizontal component of wall deformation. Once again, Meriggi et al. [19] only considered the enhancement of shear capacity and stiffness from the horizontal strips.

In terms of design specifications, vertical strips are generally excluded from the shear capacity analysis of URM walls worldwide, including in the USA, Italy, or China [20,21,22]. However, recent research has clearly demonstrated that strengthening with vertical FRP systems can increase the shear capacity and ductility of URM walls. Petersen et al. [23] investigated the shear capacity of brick walls strengthened with near surface mounted (NSM) FRP strips and reported that the vertically aligned reinforcement was an effective scheme in increasing the strength (up to 46%) and ductility of URM walls. Likewise, Konthesingha et al. [24] studied the effectiveness of strengthening URM walls with NSM FRP strips and found that the vertical strips constrained the cracking and increased failure load up to 9%. Wang et al. [25] reported on the shear strength and post-failure ductility of URM walls and concluded an increment up to 48% after strengthening with vertical SRG strips. Kaluza [26] found significant increase of capacity (up to 48%) and ductility of concrete block walls due to strengthening with vertical CFRP or GFRP strips. Increase in the shear capacity resulting from vertical fibers due to joint sliding is recognized in the specifications by ACI 549.6R [20].

This research investigates a strengthening scheme that combines an organic matrix with an impregnated glass fabric grid. In this composite, a fast-setting polyurea matrix is sprayed over a glass fiber-reinforced polymer (GFRP) grid. The resin impregnation helps with redistributing stress, preventing or delaying premature ruptures caused by localized concentrations of stress, as well as mitigating telescopic failures [20,27,28]. In our study, four concrete block walls and four clay brick walls were strengthened with discrete GFRP grid strips bonded with sprayed polyurea, with variables including strip orientation (horizontal or vertical) and strengthening configuration (single-sided or double-sided). The URM walls were tested until failure under in-plane diagonal compressive loading. The results confirmed that the discrete vertical strips effectively inhibit crack propagation along the bed joints, delay overall failure, and significantly increase the shear capacity of the URM walls. The experimental program was reported by the authors of this paper in a previous publication [29] and additional information is presented in this paper.

The novelty of this study lies in the following contributions: (A) the experimental strain behavior of the discrete polyurea strips during compression testing was investigated; (B) the increase in shear capacity of the strengthened URM walls due to discrete vertical polyurea strips was thoroughly analyzed; (C) a comparative analysis of single-sided and double-sided strengthening configurations for URM walls was performed, supplemented by additional test data from the literature.

## 2. Experimental Program

The experimental program was reported in a previous publication [29] and only the main characteristics and results are summarized here. Meanwhile, additional information is provided in this paper.

### 2.1. Test Matrix

Concrete block masonry walls with the dimensions of 1626 × 1626 × 152 mm^3^ and clay brick walls with dimensions of 1219 × 1219 × 92 mm^3^ were strengthened according to the layout depicted in Table 1. In Table 1, n denotes the retrofit scheme, concrete walls are defined as WCn, and clay brick walls are defined as WKn. The size of the concrete blocks and the clay bricks was 152 × 203 × 406 mm^3^ and 57 × 92 × 197 mm^3^, respectively. The thickness of the head and bed joints was about 9.5 mm. The concrete block wall test consisted of testing four unstrengthened control block walls and four strengthened concrete block walls with 114 mm wide GFRP grid reinforced polyurea strips. Likewise, during the clay brick walls testing phase, except for one unstrengthened control brick wall, four brick walls were also strengthened with 114 mm wide GFRP grid reinforced polyurea strips. The scheme was adopted to study the effectiveness of orientation of the strips (horizontal or vertical) and strengthening configuration (single-sided or double-sided). The spacing between the strips was kept smaller than the spacing limit described in ACI 440.7R [30]. Further details can be found in the previous publication [29].

### 2.2. Material Properties

The GFRP grids were made from individual glass cords and connected to each other by transverse cords of a smaller size. Type N mortar [31] was used to build the walls. Material characterization of the sprayed polyurea, the GFRP grid, the grid reinforced polyurea, and the masonry units are reported elsewhere [29,32], and the major mechanical property is shown in Table 2.

### 2.3. Strain Gauges Locations

**Figure 1:**
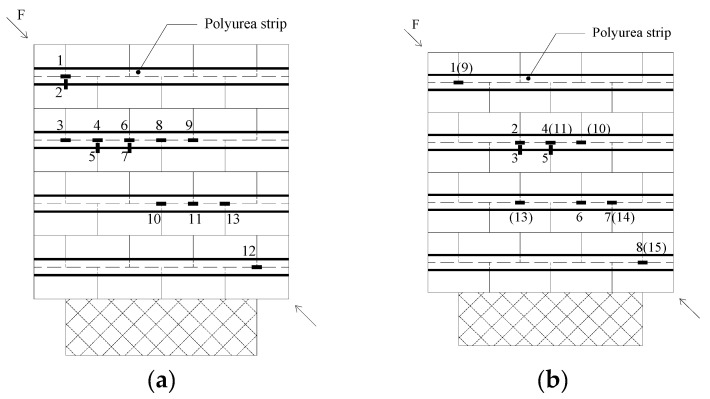

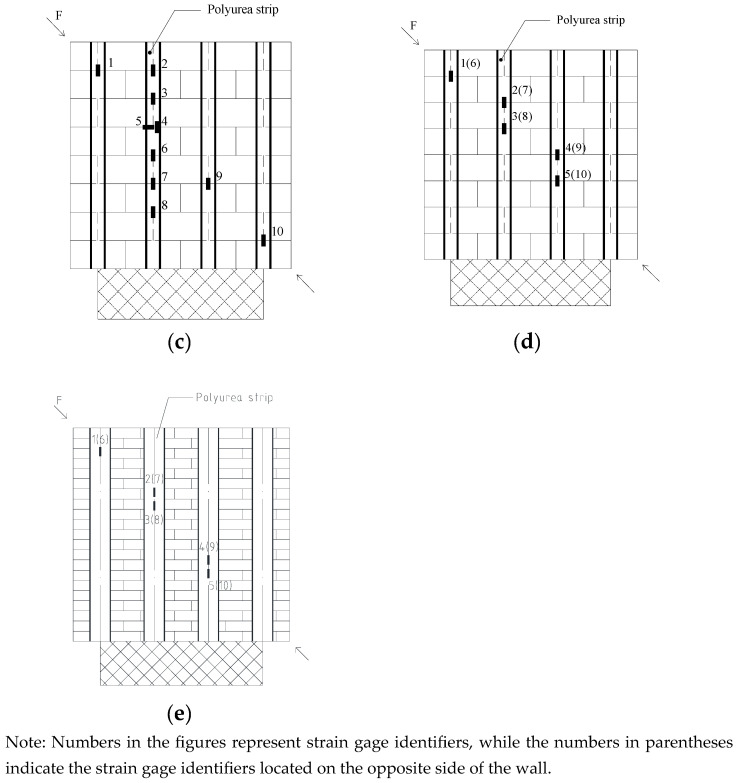
Strain gages placement: (**a**) wall WC1—single side strengthening; (**b**) wall WC2—double side strengthening; (**c**) wall WC3—single side strengthening; (**d**) wall WC4—double side strengthening; (**e**) wall WK4—double side strengthening.

Figure 1 shows the location of the strain gauges that were attached to the GFRP grid reinforced polyurea strips on both sides of the walls. Strain gauges located at the back side are indicated in parentheses. The locations of strain gauges along walls WK1–WK3 are similar to those for the counterpart concrete block walls WC1–WC3 in Figure 1. The capacity of the strain gages was 15,000 µɛ.

### 2.4. Test Setup

**Figure 2:**
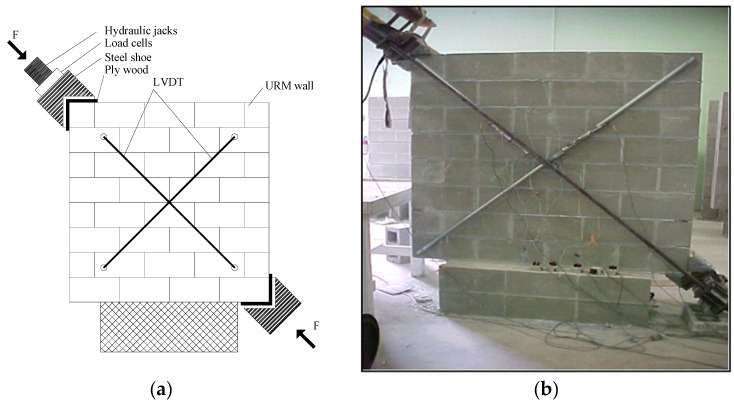
Test Setup. (**a**) schematic test setup; (**b**) test setup of one concrete block wall.

The walls were tested until failure according to the setup depicted in Figure 2. The setup was modified from its original form in ASTM E519-10 [36] and adopted by many researchers [3,37,38]. As shown in Figure 2, the walls were loaded diagonally with manually activated hydraulic jacks positioned on one corner of the walls, where two jacks were used in order to decrease the eccentricity of loading. The applied diagonal force was measured using two 450 kN capacity compression load cells placed between the hydraulic jacks and the steel shoe. Four linear variable differential transducers (LVDTs) with a gauge length of 200 mm were also installed diagonally along both sides to measure the displacements of the walls. The walls were subjected to a continuous load up to 50% of the expected maximum force, after which the load was applied in smaller increments (10–20% of the peak force) until the maximum force was reached and the test was concluded. In total, a test was completed within 30–60 min.

## 3. Experimental Results

### 3.1. Failure Mode and Failure Load of the Tested Walls

**Figure 3:**
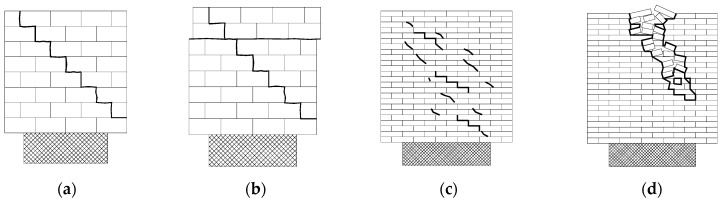
Schematic failure mode of the tested wall: (**a**) shear friction; (**b**) shear friction + shear slide; (**c**) shear friction + diagonal tension; (**d**) diagonal compression.

**Figure 4:**
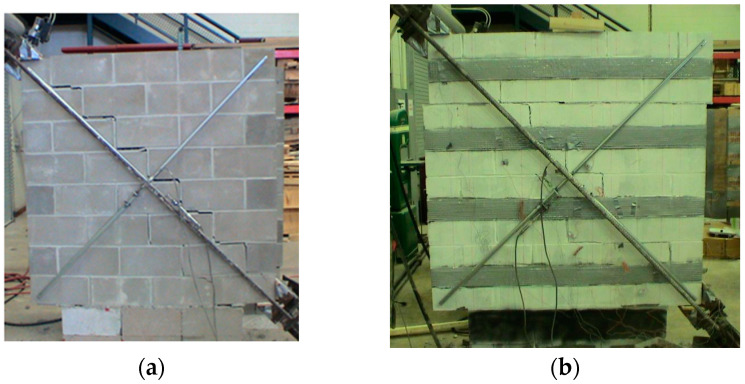

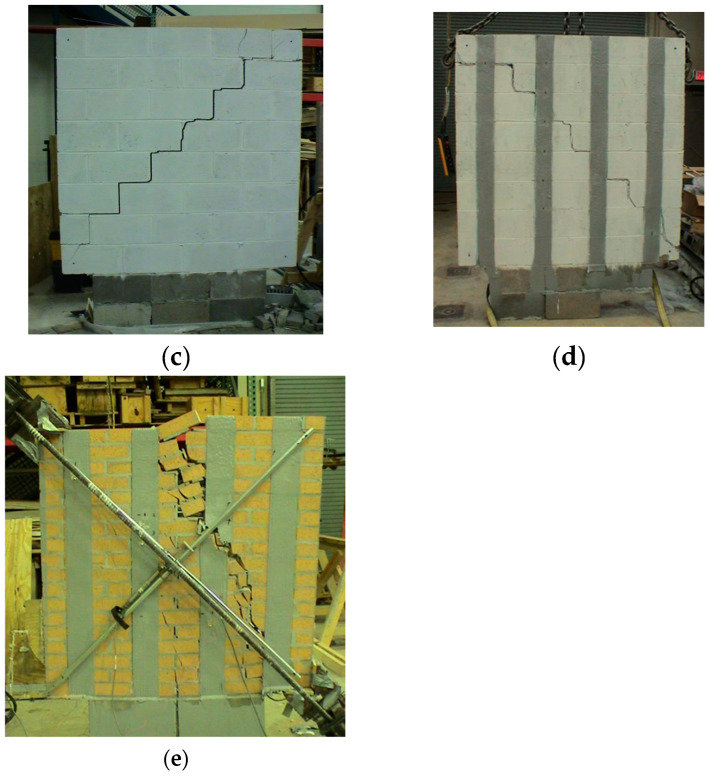
Failure mode of the tested walls: (**a**) typical shear friction failure; (**b**) wall WC 2; (**c**) wall WC 3; (**d**) wall WC 4; (**e**) wall WK 4.

As shown in Table 3, four types of failure modes are observed in the test and are schematically shown in Figure 3. The unstrengthened control walls failed due to diagonal stepped cracks (Figure 4a). For wall WC2 with double-sided strengthening, diagonal cracks were observed before the wall failed due to a sliding crack along the horizontal bed joint (Figure 4b). For concrete block walls with discrete vertical strips, the walls failed due to a stepped shear friction crack along the loading diagonal (Figure 4c,d). For clay brick wall WK4, a crack along the joints was prevented due to the double-sided strengthening strips, and the wall failed due to the failure of the bricks under diagonal compression (Figure 4e). Additional information can be found elsewhere [29].

### 3.2. Experimental Strains of the Polyurea Strips

**Figure 5:**
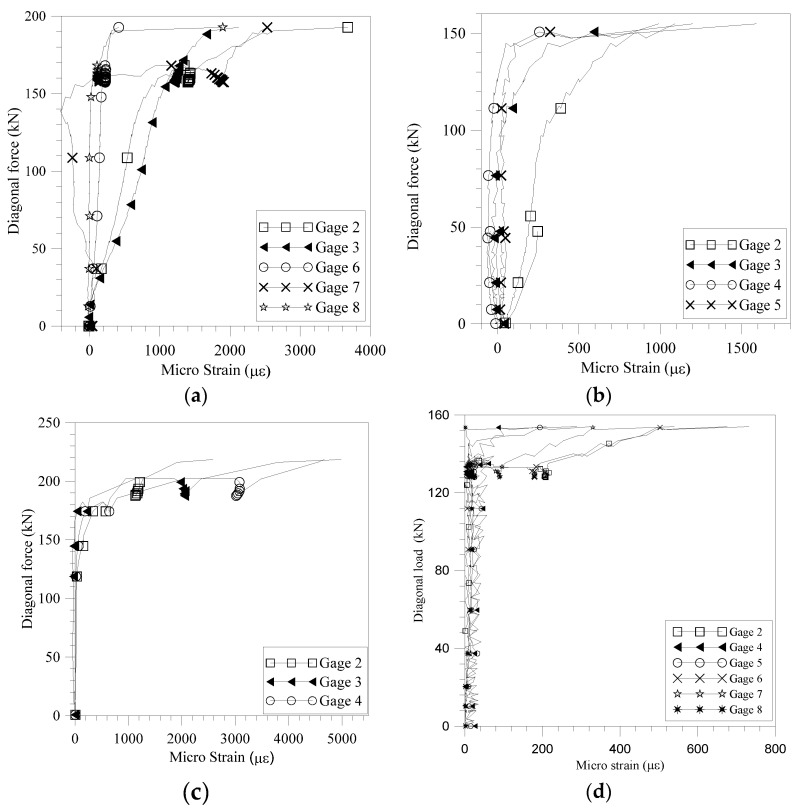
Experimental strain of polyurea strips: (**a**) wall WC2; (**b**) wall WC3; (**c**) wall WC4; (**d**) wall WK4.

The maximum strains of the strips are shown in Table 4 and summarized as follows:

1. Walls WC1, WK1, WK2, and WK3: The recorded strains are negligible, and therefore, are not included in Table 4. This aligns with what was reported by Jing et al. [39]. In their test, the ratio of the recorded maximum strain to the ultimate limit of the CFRP plate at failure for the URM walls was 8.94%; however, a significant increase in shear capacity was achieved. The reason is that other than the shear force taken by the FRP itself, the restraining effect of the FRP resulted in a significant increase in the shear capacity. Therefore, the restraining effect, due to the 7 mm thick sprayed polyurea strips in the test, was responsible for the greater increase of shear capacity of the URM walls even though the recorded strain of the strips was low.
2. Wall WC2: Table 4 and Figure 1b and Figure 5a illustrate that the recorded strains on the FRP strips are negligible when the strain gauges are positioned away from the diagonal cracks. At failure, the strain readings from gauges 2/3 and 4/5 are nearly identical, suggesting that the horizontal strips were subjected to significant tension in the vertical direction at the locations where the stepped cracks intersected. Additionally, strain readings from gauges 4, 7, and 8 on the front side, as well as from gauges 11, 14, and 15 on the back side, show that the strains in the strips at corresponding locations on each side are significantly different.
3. Wall WC3: Table 4 and Figure 1c and Figure 5b show that the upper portion of the vertical strips is under significant tension. Meanwhile, the strain of gage 5 tells that the horizontal tension recorded in the vertical strips helped inhibit cracks along the head joints.
4. Wall WC4: High strains in the vertical direction, as shown in Figure 1d and Figure 5c, indicate that the vertical strips contributed to constraining dilation along the bed joints. Furthermore, the variation in strain readings from gauges 3, 4, and 5 on the front side, and from gauges 8, 9, and 10 on the back side, reveals that, at failure, the strains measured in the strips at corresponding locations on each side differ significantly (Table 4 and Figure 1d).
5. Wall WK4: Shear cracks along the mortar joints were not observed, which can be attributed to the clamping mechanism provided by the vertical strips. The strains recorded along the strips from both sides at the maximum diagonal load were relatively small (Table 4 and Figure 1e and Figure 5d). The wall exhibited considerable post-peak load ductility, as strains in the strips increased significantly, while the diagonal load remained nearly constant or experienced only a small decrease.

Overall, the test results demonstrate that no premature debonding of the discrete polyurea strips occurred during the experiments. This is noteworthy, especially considering the absence of mechanical anchorages at the end zones between the concrete block or the brick substrate and the polyurea strips. The fact that the polyurea strips remained bonded without additional anchorage suggests that, for GFRP-reinforced polyurea systems, the need for supplementary anchorage at the strip ends may be unnecessary, enhancing the practicality and efficiency of this strengthening technique.

Furthermore, the recorded strains in the double-sided strengthened walls showed a clear difference between the strengthening materials applied on each side. This asymmetry in strain response indicates that the strengthening contribution from each side was not equal, with one side potentially providing a more significant effect on the overall performance of the wall. Such a difference in contribution could arise from factors such as material properties, the quality of bonding, or stress distribution during loading. It is important to emphasize that no significant out-of-plane deformation or bending was observed during the diagonal compression tests, which suggests that the overall structural behavior of the walls remained stable and the strengthening systems functioned as intended.

Given these observations, when analyzing the shear capacity of the URM walls strengthened with double-sided polyurea systems, it is crucial to apply a reduction factor. This factor would account for the asymmetrical performance of the system and the potential effects of eccentricity that could result from the unequal contribution of the strengthening materials on each side. Incorporating this reduction factor into the design and analysis would lead to more accurate predictions of wall performance under real-world loading conditions, ensuring the robustness and reliability of the strengthening technique.

### 3.3. Shear Strain of the URM Walls

Shear strains were computed according to ASTM E519 [36] as follows:

(1) $$\gamma =\frac{\Delta S+\Delta L}{g}$$

where ΔS and ΔL are the measured shortening and elongation diagonally and *g* is the LVDTs gage length.

The pseudo-ductility of the walls were evaluated per the following:

(2) $$\mu =\gamma_{u}/\gamma_{y}$$

In general, $\gamma_{u}$ is the shear strain registered at 20% off the peak shear stress at the post peak stage [7,40], and $\gamma_{y}$ is the shear strain corresponding to 70% of the maximum shear stress in the ascending loading branch [7,41] or corresponding to the peak shear stress [42,43]. In this paper, $\gamma_{y}$ was calculated at the peak shear stress. Table 3 shows that, for both the concrete block and the clay brick walls, the strengthened walls demonstrated a larger post peak-load pseudo-ductility capacity than the plain control walls. Furthermore, the URM walls strengthened with vertical strips generally achieved greater pseudo-ductility, with the exception of walls WC1 vs. WC3 in Table 3.

### 3.4. Evaluation of the Test Results

Results from other in-plane diagonal compression tests using the URM walls strengthened with FRP, FRCM, or SRG are summarized in this section for further analysis.

### 3.5. Effect of Strips Layout on the Shear Strengthening of URM Walls

ACI 440.7R [30] specifies a maximum clear spacing of 400 mm between externally bonded strips in fiber-reinforced polymer (FRP) systems. In the tests reported in Table 3, the clear spacing between the horizontal strips in the concrete block and clay brick walls was 305 mm and 203 mm, respectively. As shown in Table 3, walls WC2 and WK2 failed due to shear sliding along the unstrengthened bed joints, indicating that the application of only horizontal strips was insufficient to prevent sliding failure. This finding aligns with previous studies by Mahmood and Ingham [3] and Konthesingha et al. [24], which reported similar outcomes.

Furthermore, these walls exhibited lower pseudo-ductility compared to those strengthened with other configurations, as seen in Table 3. This observation is particularly significant when considering seismic retrofit applications, where pseudo-ductility is a key design objective. In such cases, where both sides of the URM walls are strengthened to enhance energy dissipation and prevent brittle failure, achieving higher pseudo-ductility is crucial. Therefore, the results suggest that a clear spacing of less than 400 mm may be necessary for such strengthening schemes to ensure the desired level of ductility and the overall performance for seismic applications.

The tension strain in the GFRP strips was consistently recorded in this study, regardless of whether the URM walls were strengthened with horizontal or vertical strips. This suggests that grid strengthening with both horizontal and vertical strips could be an effective scheme for enhancing the shear capacity and ductility of URM walls. This approach is supported by the literature [18,20] and the test results of walls S12GH, S12GV, and S12GHV, as shown in Table 5. These results demonstrate that the capacity and ductility of walls strengthened with orthogonal strips were greater than those strengthened with only horizontal or vertical strips.

Whether the walls were strengthened with discrete strips or full-surface strengthening, the results indicate that vertical strengthening significantly improved both the strength and ductility of the URM walls (Table 5). Additionally, increasing the axial stiffness of the FRP material led to the enhanced shear capacity of the walls, as evidenced by comparing WC3 with WC4 and WK3 with WK4 in Table 3. Notably, when the total cross-sectional area of the strengthening materials remained constant, discrete strip strengthening resulted in a greater increase in shear capacity compared to full-surface strengthening (see comparisons of S4H vs. S12GH and S4V vs. S12GV in Table 5).

Further, when the same amount of strengthening material was used, the results suggest that greater ductility could be achieved with vertical strips instead of horizontal ones. This is observed in the comparison of walls S4V with S4H, S12GV with S12GH, and WTC 5 with WTC 2 in Table 5, and the walls presented in Table 3. Moreover, since discrete vertical strips can effectively induce compression failure in URM walls (see Figure 4e), additional strengthening materials may not provide further benefit in terms of performance.

Ferretti and Mazzotti [44] reported that the central region of masonry panels experiences higher tensile stresses during diagonal compression tests, particularly at the onset of cracking. As a result, it is critical to ensure sufficient strengthening in the central regions, even for walls that did not directly fail in this area during testing. This consideration is essential for optimizing the overall performance of the strengthened URM walls and preventing premature failure in critical regions.

### 3.6. Effective Strain of Polyurea Strips at Failure of the Strengthened URM Walls

As reported in the literature, thicker and stiffer FRP composites are more susceptible to premature debonding failure. Valluzzi et al. [45] observed that less stiff FRP materials were more effective for increasing the ultimate strength and stiffness of masonry panels. Additionally, the effective strain of FRP strips has been found to be inversely proportional to their axial stiffness, $\rho E_{frp}$, with FRP materials exhibiting higher axial stiffness tending to debond at lower strains [16,46]. For FRCM-masonry joints, Ceroni and Salzano [47] and Meriggi et al. [19] found that the failure strain due to slippage and debonding decreased as axial stiffness, $A_{f}E_{f}$, increased.

However, for the URM walls strengthened with vertical polyurea strips, results from this study, as shown in Table 4, indicate that the maximum strain in the strips of wall WC4 is significantly greater than that of wall WC3. This can be attributed to the increased overall integrity of the URM walls when vertical strips are applied on both sides. This dual-sided strengthening delayed shear friction failure compared to single-sided strengthening, thereby enhancing the effective strain of the strengthening strips. A similar trend was observed for brick walls strengthened with NSM FRP strips, where the maximum strain in the vertical strips of double-sided strengthening was generally greater than that in single-sided strengthening (see walls V2 and V4A, V4B in Table 5).

Moreover, Table 4 shows that, when discrete strips with the same axial stiffness are used, the maximum strain in the vertical strips is generally greater than that in the horizontal strips. This suggests that it may be reasonable to adopt different allowable strain limits for horizontal and vertical strengthening schemes when evaluating the shear contribution of the strips.

**Figure 6:**
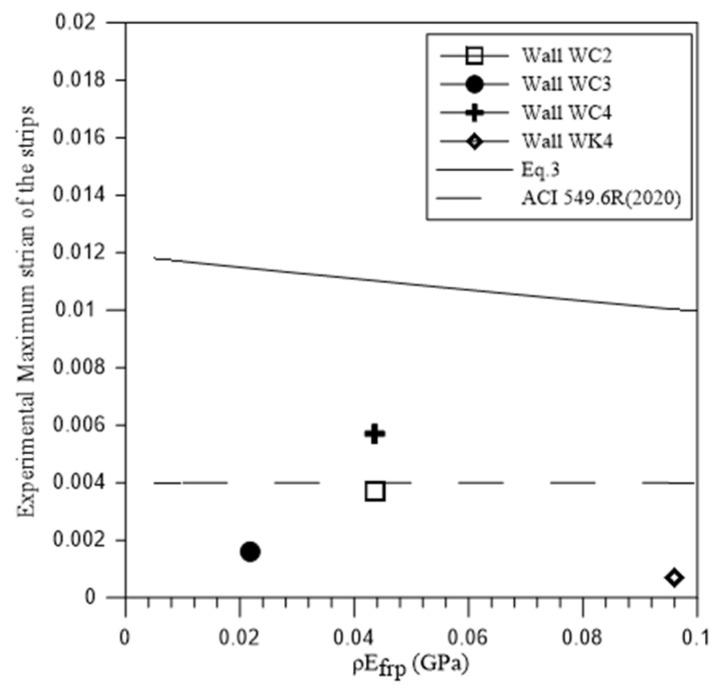
Strain of polyurea strips [20].

The typical model to predict the effective strain of an FRP sheet bonded to masonry was developed by Triantafillou [16] (when $\rho_{h}E_{frp}$ is less than 1 GPa)

(3) $$\varepsilon_{frp,e}=0.0119-0.0205\left(\rho_{h}E_{frp}\right)+0.0104{\left(\rho_{h}E_{frp}\right)}^{2}$$

where, $\rho_{h}$ and $E_{frp}$ are the reinforcement ratio and the elastic modulus of FRP strips, respectively. A comparison between the recorded tension strain of polyurea strips and Equation (3) in Figure 6 indicates that this model overestimated the effective strain in this test.

In the diagonal compression test of the URM walls strengthened with discrete polyurea strips, the stress condition of the strips is significantly influenced by the various cracking patterns of the walls. The bonding mechanism of the strengthening material in this context differs from that observed in typical bond tests. Furthermore, no debonding of the polyurea strips was observed during the tests. As a result, models based on standard bonding tests may not accurately predict the effective strain of the strips during the compression test and may not be directly applicable for structural analysis of the strengthened URM walls without appropriate modifications.

Code specifications typically define a constant effective strain for horizontal strips [20,22]. However, it appears that the effective strain of the horizontal strips is influenced by several factors, including the reinforcement ratio, layout of the strengthening materials, and other variables (see Table 4). Therefore, the fixed effective strain value as specified by ACI 549.6R [20] may oversimplify the actual behavior (Figure 6). Moreover, assuming that each horizontal strip reaches the limit strain specified in the code could lead to an overestimation of the contribution of the horizontal strengthening materials to the shear capacity of the strengthened walls.

### 3.7. Contribution of Discrete Vertical Strips to Shear Capacity of the URM Walls

Table 5 demonstrates that the use of discrete vertical FRP strips and SRG strips resulted in a significant increase in shear capacity, with improvements of up to 46% for wall V4B [23], 48% for wall S12GV [25], and 221% for wall WTC 5 [3].

As shown in Table 4, significant tension was recorded in the discrete vertical strips when the walls failed due to cracking along the joints. This suggests that the vertical strips played a crucial role in carrying significant tensile forces resulting from the horizontal shear. Initially, the tension in the strips was minimal when the diagonal load was relatively low. However, as the load approached the failure point, the tension in the strips increased substantially (see Figure 5). This increase in tension contributed to compressive forces along the bed joints of the URM walls. For the URM walls strengthened with polyurea strips that failed due to cracking along the bed joints, the shear strength contribution from the vertical strips can be calculated as follows:

(4) $$\Delta V=\Delta V_{1}+\Delta V_{2}$$

where $\Delta V_{1}$ is the shear strength due to the increased compressive load at the bed joints that resulted from the vertical strips and $\Delta V_{2}$ is the contribution from the strips itself. Similar to the approach in Silva et al. [29], the shear strength due to the increased compressive stress could be calculated as follows:

(5) $$\Delta V_{1}=\frac{\mu E_{frp}\varepsilon_{frp}A_{frp}}{1-\mu \tan\alpha }$$

where, $A_{frp}$ and $E_{frp}$ are the total cross-sectional area and elastic modulus of the vertical strips, respectively, $\varepsilon_{frp}$ is the effective strain of the vertical strips, μ is the shear friction coefficient, and α is the angle between the loading diagonal and horizontal direction. The shear contribution of the vertical strips is as follows [29]:

(6) $$\Delta V_{2}=E_{frp}\varepsilon_{frp}A_{frp}/\sqrt{3}$$

Therefore, the total contribution of the vertical strips can be calculated as follows:

(7) $$\Delta V=\left(\frac{\mu }{1-\mu \tan\alpha }+\frac{1}{\sqrt{3}}\right)E_{frp}\varepsilon_{frp}A_{frp}$$

In this study, a shear friction coefficient *µ* of 0.5 and *α* of 45° were considered in the test analysis. Table 6 shows that, for the URM walls strengthened with vertical strips that failed due to the stepped shear-friction cracks, Equation (7) gives a reasonable prediction of the shear capacity. One reason for the discrepancy between the experimental and theoretical capacity of wall WC3 is that the maximum strain of polyurea strips may not be caught during the test, as only a limited number of strain gauges were deployed.

**Figure 7:**
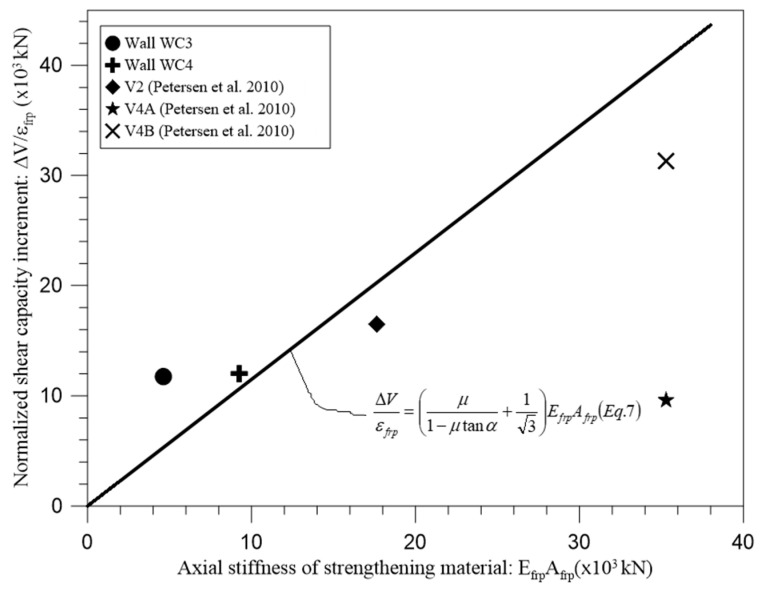
Shear capacity increment of URM walls due to discrete vertical strips [23].

Moreover, with addition of test results from Petersen et al. [23], Figure 7 indicates that Equation (7) can provide a reasonable estimation of the shear capacity increment of the URM walls strengthened with discrete vertical strips (GFRP grid strips or CFRP strips) that failed mainly due to stepped joint cracks during diagonal compression test.

### 3.8. Comparison of Double-Sided Strengthening with Single-Sided Strengthening

In general, the single-sided strengthening of URM walls may introduce structural asymmetry, potentially causing out-of-plane bending and a significant strength degradation. ACI 549.6R [20] and Meriggi et al. [19] recommend applying a reduction factor of 0.7 for single-sided strengthening with FRCM to account for eccentricity, whereas a factor of 1.0 is applied for double-sided strengthening with FRCM.

Table 3 reveals that, for the URM walls strengthened with polyurea strips that failed due to cracking along the joints, the shear capacity increment factor for double-sided strengthening over single-sided strengthening ranges from 1.18 to 3.66. This indicates that the asymmetry associated with single-sided strengthening is influenced by the layout of the strengthening scheme, and increasing the axial stiffness of the strips may mitigate the negative effects of structural eccentricity (see walls WC1–WC2 and WK1–WK2 in Table 3). Regarding the orientation of the strengthening materials, single-sided strengthening with vertical strips caused a more pronounced negative impact compared to horizontal strips (see walls WC1–WC2 and WC3–WC4 in Table 3).

Table 7 demonstrates that the increment factor for shear capacity with double-sided strengthening over single-sided strengthening ranges from 1.12 to 3.18 for full surface strengthening, and from 1.07 to 2.77 for discrete strip/bar strengthening. Therefore, a single constant reduction factor, as stated in ACI 549.6R [20], may not adequately cover all cases, as many factors—such as strengthening configuration, material type, masonry typology, and geometry—contribute to performance differences between single-sided and double-sided strengthening systems. Furthermore, Table 3 and Table 7 indicate that there is no significant difference in shear capacity increment when organic or inorganic matrices are employed.

The results presented in Table 3 and Table 7 show that the increment factor for shear capacity of double-sided strengthening over single-sided strengthening in many tested walls is less than 2.0. Consequently, the average effective strain in double-sided strengthening materials should be lower than in single-sided strengthening, and a reduction factor should be introduced during the design phase to account for this situation. This is comparable to findings by Yang et al. [49], which demonstrated that the strengthening effect did not increase linearly with the addition of textile layers and that the strengthening efficiency of the URM walls decreased as more layers of basalt were employed.

Given that detailed strain data for strengthening materials at failure is often not reported in the literature, and the minimum shear capacity increment factor observed is around 1.2 (Table 3 and Table 7), a reduction factor of 0.6 could be used for design purposes.

Furthermore, as shown in Table 3 and Table 7, the pseudo-ductility of the strengthened URM walls increased compared to control walls. While the shear capacity of walls with double-sided strengthening was greater than that of walls with single-sided strengthening, the ductility of double-sided strengthened walls could be lower when the walls fail predominantly due to cracking along the joints. This observation is supported by the tests reported by Marcari et al. [5] and Mahmood and Ingham [3] in Table 7, and the results of walls WC3–WC4 and WK1–WK2 in Table 3.

### 3.9. Failure Mode of the URM Walls Strengthened with Vertical GFRP Strips

The experimental results of the URM walls strengthened with vertical GFRP schemes are further compared in Table 6, where for Wall 6 [48] and COW 9 [37] only the vertical strengthening is included. Elastic modulus of concrete block and clay brick masonry was estimated with $E_{m}=900\;f_{m}^{\prime }$ and $E_{m}=700\;f_{m}^{\prime }$, respectively, according to MSJC standard [50]. It is shown in Table 6 that, for the strengthening of URM walls with GFRP, with the increase of $\rho \;E_{frp}/E_{m}$, the failure mode of the wall changed from stepped joint failure (shear friction) to combined failure (shear friction + tension of brick) or compression failure of the masonry units. However, the exact boundary between the different failure modes could not be precisely determined in this paper as the failure modes is also related to the layout and mechanical property of the strengthening materials, and many other factors. Furthermore, since the diagonal crushing is achieved, additional strengthening may not be necessary, which imposes the limit for strengthening.

Meanwhile, when vertical strips are used for shear strengthening, typically it is required to check the toe crushing by the code [20,21]. However, as seen from this study, for the strengthening of clay brick walls, instead of toe crushing, diagonal compression outside of the compression toe was observed (WK4 in Figure 4e and Table 3). As it is reported, during the diagonal compression test, the principal compression stress is around three times that of the tensile stress along the loaded diagonal [51]. Therefore, except for toe crushing, compression failure of the walls should also be checked.

### 3.10. Design Recommendations

For the shear strengthening of URM walls with discrete polyurea strips, the following recommendations should be considered:

1. Concrete Block Walls: when only discrete horizontal strips are used for shear strengthening, it is advisable to cover each of the bed joints to effectively prevent shear sliding failure along the bed joints.
2. Double-Sided Strengthening: for strengthening of the URM walls with horizontal strips, assuming the contribution of double-sided strengthening is equal to twice that of single-sided strengthening may lead to an overestimation of the shear strength increment; therefore, a reduction factor of 0.6 should be considered for design purposes until more experimental data is available.
3. Discrete Vertical Strips vs. Full Surface Strengthening: since discrete vertical strips have been found to be more efficient than full surface strengthening when the same amount of strengthening fabric or grid is used, it is recommended to apply discrete vertical strips instead of full surface strengthening for improved shear capacity and cost-effectiveness.

## 4. Conclusions

This research provides valuable insights into the in-plane strengthening of unreinforced masonry (URM) walls using discrete glass fiber-reinforced polymer (GFRP) grid strips bonded with sprayed polyurea. This study focused on the effects of strengthening orientation (horizontal and vertical), the configuration (single-sided and double-sided), and the interplay between material properties and structural performance under diagonal compression loading. The findings underscore the significant contributions of vertical GFRP strips to both the shear capacity and pseudo-ductility of the URM walls. The following conclusions can be drawn from this research program:

- Vertical Strengthening Efficacy: The application of vertical GFRP strips effectively constrained the development of bed joint cracking and delayed the overall failure of the URM walls. This resulted in a notable improvement in shear capacity and pseudo-ductility compared to unstrengthened walls or those strengthened only with horizontal strips.
- Enhanced Effective Strain: The discrete vertical strips demonstrated a higher effective strain compared to horizontal strips, particularly in walls failing due to shear friction cracking. This suggests that greater strain allowances can be adopted when considering the contribution of vertical strips to the overall shear capacity of the strengthened URM walls.
- Predictive Accuracy of Shear Models: the preliminary model proposed in this study reasonably predicted the shear capacity increments due to the inclusion of vertical GFRP strips, highlighting the potential for refined analytical tools in structural design applications.
- Asymmetrical Behavior in Double-Sided Strengthening: Despite the absence of out-of-plane bending, the strain responses differed significantly between the two sides of double-sided strengthened walls. This asymmetry emphasizes the need for reduction factors to accurately reflect the unequal contributions of each side in design calculations.
- Superiority of Vertical Strips in Pseudo-Ductility: vertical strengthening schemes consistently achieved higher pseudo-ductility compared to horizontal ones, reinforcing their suitability for seismic retrofitting where energy dissipation and deformation capacity are critical.

In conclusion, this study advances the understanding of strengthening URM walls, highlighting the potential of discrete vertical GFRP strips for enhancing seismic performance. It advocates for the broader adoption of this approach in seismic retrofitting practices, offering valuable insights into its effectiveness. However, this study’s findings are primarily based on diagonal compression tests, which involve combined compression–shear stress states. As such, further experimental validation is needed to explore other loading scenarios and stress conditions. Future research should aim to fully characterize the response of the strengthened URM walls under various loading conditions and validate the design recommendations derived from this study.

## Figures and Tables

**Table 1:** Strengthening schemes.

Wall	Directional Layout	Sides Being Strengthened
WC0a	--	--
WC0b	--	--
WC0c	--	--
WC0d	--	--
WC1	horizontal	single
WC2	horizontal	double
WC3	vertical	single
WC4	vertical	double
WK0	--	--
WK1	horizontal	single
WK2	horizontal	double
WK3	vertical	single
WK4	vertical	double

**Table 2:** Material properties.

	GFRP Gird	Plain Polyurea	GFRP Grid Reinforced Polyurea (Based on the Cross-Section of Grid)	Concrete Block	Clay Brick	Mortar
Tensile or compressive strength (MPa)	587-t	7-t	736-t	16.8-c	13.2-c	5.67-c
Elastic modulus (GPa)	37-t	0.2-t	37.7-t	15.1-c	11.2-c	2.8-c
Ultimate strain (%)	1.8	43.8	2.3	--	--	--
Test standard followed	ASTM D3039 [33]	ASTM D3039 [33]	ASTM D3039 [33]	ASTM C1314 [34]	ASTM C1314 [34]	ASTM C109 [35]

t—tension; c—compression.

**Table 3:** Experimental results.

Wall	Failure Mode	Failure Diagonal Load F (kN)		^(b)^ Increase Capacity (%)	^(c)^ Increment Factor	$\gamma_{y}$ (×10^−3^) (1)	$\gamma_{u}$ (×10^−3^) (2)	Pseudo- Ductility (2)/(1)
WC0a	SF	108.1	^(a)^ 128.3	--	--	--	--	--
WC0b	SF	116.5		--	--	0.28	0.81	2.9
WC0c	SF	153.8		--	--	0.27	2.92	8.4
WC0d	SF	134.9		--	--	0.34	1.27	3.74
WC1	SF	191.3		^(x)^ 49.1	--	0.12	1.35	11.25
WC2	SF + SS	238.0		85.4	^(y)^ 1.73	0.30	1.44	5.33
WC3	SF	154.8		20.6	--	0.46	4.88	10.6
WC4	SF	225.1		75.4	3.66	0.40	2.58	6.45
WK0	SF	81		--	--	1.62	4.25	2.61
WK1	SF + SS	149		83.8	--	0.089	1.25	14.4
WK2	SS	161.5		99.3	1.18	0.209	1.70	8.1
WK3	SF + tension	132.6		63.7	--	0.030	0.55	18.3
WK4	compression	148.6		83.5	--	0.227	16.05	70.7

SF: shear friction; SS: shear slide. ^(a)^ The average peak force registered in the unstrengthened test walls WC0a, WC0b, WC0c, and WC0d is 128.3; standard deviation is 17.54, and coefficient of variation is 0.14. ^(b)^ Increase in capacity in relation to the average force of unstrengthened walls. ^(x)^ Example of increased capacity is 49.1% = (191.3 − 128.3)/128.3 or 85.4% = (238.0 − 128.3)/128.3. ^(c)^ Increment factor defined as ratio of shear capacity increment due to double-sided strengthening over single-sided strengthening. ^(y)^ Example increment factor: 1.73 = 85.4/49.1 or 3.66 = 75.4/20.6.

**Table 4:** Recorded strain of the polyurea strips at maximum diagonal load for the URM walls.

	Wall WC2 (*µɛ*)	Wall WC3 (*µɛ*)	Wall WC4 (*µɛ*)	Wall WK4 (*µɛ*)
Gage 1	325	128	603	126
Gage 2	3677	1091	2861	675
Gage 3	2122	990	5689	209
Gage 4	414	1592	4983	45
Gage 5	94	1199	−132	102
Gage 6	2363	−121	2369	324
Gage 7	2530	−20	47	731
Gage 8	1896	−67	2179	539
Gage 9	−381	−67	−254	15
Gage 10	−88	−100	1119	288
Gage 11	−552	--	--	--
Gage 12	--	--	--	--
Gage 13	−14	--	--	--
Gage 14	−64	--	--	--
Gage 15	502	--	--	--

**Table 5:** Shear strengthening of URM walls with vertical and horizontal schemes in the literature.

Reference	Wall	Strengthening Schemes	Failure Mode	Recorded Maximum Strain (*µɛ*)	Failure Load F (kN)	Estimated URM Walls ^$^ (kN)	Load Increase (%)	Ductility #
Petersen et al. [23]	V2	Two vertical NSM CFRP strips on one side	N/S	3100	160	125	28	N/A
	V4A	Two vertical NSM CFRP strips on each side	N/S	4000	210	172	22	N/A
	V4B		N/S	6242	205	140	46	N/A
	H4A	Two horizontal NSM CFRP strips on each side	N/S	8900 *	264	251	5	N/A
	H4B		N/S	1600	185	183	2	N/A
	V2H2A	Two horizontal NSM CFRP strips on one side and two vertical on the other side	N/S	3590 (horizontal) /3800 (vertical)	206	177	16	N/A
	V2H2B		N/S	9850(vertical)	158	120	32	N/A
Wang et al. [25]	URM1	--	ST	--	114	--	--	--
	URM2	--	ST	--	124	--	--	--
	S4H	Full surface horizontal SRG	TF	N/A	204	--	72	6.65
	S4V	Full surface vertical SRG	TF	N/A	155	--	30	7
	S12GH	Three horizontal SRG strips	TF, TC	N/A	231	--	94	2.67
	S12GV	Three vertical SRG strips	TF	N/A	175	--	48	7.90
	S12GHV	Three horizontal and three vertical SRG strips	TC	N/A	263	--	121	8.42
	S4GHV		TC	N/A	210	--	78	14.93
Mahmood and Ingham [3]	AP6	--	SS + SF	--	36	--	--	13.8
	AP7	--	SS + SF	--	35	--	--	6.5
	WTC2	Three horizontal CFRP strips	SS + SF	N/A	92	--	166	6.6
	WTC3	Three vertical and three horizontal CFRP strips	SS + SF	N/A	98	--	183	10.3
	WTC5	Three vertical CFRP strips	SS + SF	N/A	111	--	221	10.9

N/A = not available; ST = combined sliding along mortar joint and cracking in masonry units; SS = shear slide; SF = shear friction; TF = TRM (or SRG) failure; TC = toe crushing; N/S = not specified in original resource; NSM = near surface mounted; SRG = steel reinforced grout; * due to large bending after cracking; # defined as ultimate shear strain over yield shear strain; ^$^ given in original resources.

**Table 6:** Experimental and theoretical shear capacity increment due to vertical GFRP schemes.

Wall	$A_{frp}$ (mm^2^)	$E_{frp}$ (GPa)	$E_{m}$ (GPa)	$\rho^{*}\frac{E_{frp}}{E_{m}}$ (%)	Failure Mode	Experimental			Calculated ΔV (Equation (7)) (kN)
						V (kN)	ΔV (kN)	Maximum $\varepsilon_{frp}$ (*µɛ*)	
Wall 6 ^I^ [48]	143.2	83.4	15.1	0.32	SF	--	--	--	--
COW 9 ^I^ [37]	36.1	83.1	15.1	0.08	SF	--	--	--	--
WC3 ^I^	2895 ^#^	1.59	15.1	0.12	SF	109.4	14.6	1592	10.9
WC4 ^I^	5791 ^#^	1.59	15.1	0.25	SF	159.2	64.9	5689	81.3
WK3 ^II^	2895 ^#^	1.59	11.2	0.37	Shear friction + Tension	--	--	--	--
WK4 ^II^	5791 ^#^	1.59	11.2	0.73	Compression	--	--	--	--

^#^ Based on cross section of polyurea strips reinforced with GRRP grid; * $\rho =\frac{A_{frp}}{A_{W}}$; ^I^ $A_{W}$ = 1626 × 152 = 0.247 m^2^; ^II^ $A_{W}$ = 1219 × 92 = 0.112 m^2^.

**Table 7:** Experimental results of other diagonal compression tests in the literature.

Reference	Wall	Strengthening Type and Material	Sides Being Strengthened	Failure Mode ^#^	Failure Capacity (kN)	Failure Shear Stress (MPa)	Averaged Capacity or Shear Stress	Increased Shear Capacity (%)	Increment Factor ^$^	Pseudo-Ductility
Parisi et al. [41]	P1	--	None	S-SC	--	0.21	0.22	--	--	1.4
	P2	--	None	S-SC	--	0.19				3.6
	P3	--	None	S-SC	--	0.27				1.9
	PR1	FS + IMG	Single	S-SC	--	0.45	0.43	95.5 ^&^	--	2.2
	PR2	FS + IMG	Single	S-SC	--	0.41				2.7
	PRR1	FS + IMG	Double	S-SC	--	0.71	0.70	218.2	2.28 **	5.2
	PRR2	FS + IMG	Double	S-SC	--	0.68				5.5
Marcari et al. [5]	UPD1	--	None	SS	--	0.40	0.39	--	--	4.4
	UPD2	--	None	SS	--	0.37				4.5
	RPS2	FS + BTRM	Single	SS	--	0.52	0.52	33	--	8.5
	RPS3	FS + BTRM	Single	SS	--	0.53				12.9
	RPD1	FS + BTRM	Double	DF	--	0.62	0.62	59	1.79	5.8
	RPD2	FS + BTRM	Double	DF	--	0.63				7.5
Giaretton et al. [6]	UR2-1	--	None	SS	121.2	--	99.2	--	--	N/A
	UR2-2	--	None	SS	71.9	--				N/A
	UR2-3	--	None	SS	104.6	--				N/A
	R2S-1	FS + TRM	Single	DF	181.1	--	148	49.2	--	N/A
	R2S-2	FS + TRM	Single	DF	142.7	--				N/A
	R2S-3	FS + TRM	Single	DF	125.6	--				N/A
	R2S-4	FS + TRM	Single	DF	116.7	--				N/A
	R2S-th	FS + TRM	Single	DF	173.7					N/A
	R2d-1	FS + TRM	Double	DF	238.0	--	254.8	156.8	3.18	N/A
	R2d-2	FS + TRM	Double	DF	255.9	--				N/A
	R2d-3	FS + TRM	Double	DF	270.4	--				N/A
Cheng et al. [7]	W-U-1	--	None	S-SC	84.00	--	77.57	--	--	1.0
	W-U-2	--	None	S-SC	71.13	--				1.0
	W-SF-1	Diagonal FRP Strips	Single	S-SC	202.05	--	193.18	149	--	3.8
	W-SF-2		Single	S-SC	184.30	--				4.1
	W-DF-1	Diagonal FRP Strips	Double	TC	220.80	--	227.15	192	1.29	4.7
	W-DF-2		Double	TC	233.50	--				4.3
	W-SC1-1	FS + TRC	Single	S-SC	229.00	--	221.42	185	--	7.4
	W-SC1-2	FS + TRC	Single	S-SC	213.83	--				4.1
	W-DC1-1	FS + TRC	Double	TC	218.54	--	238.27	207	1.12	9.3
	W-DC1-2	FS + TRC	Double	TC	258.00	--				14.2
Prota. et al. [42]	P#1	--	--	S	--	0.22	0.24	--	--	2.2
	P#2	--	--	S-T	--	0.35				--
	P#3	--	--	S	--	0.21				2.4
	P#4	--	--	S	--	0.19				3.0
	PT#3	FS + CMG	Single	S	--	0.50	0.42	75	--	3.2
	PT#4	FS + CMG	Single	S, O	--	0.34				3.7
	PS#3	FS + CMG	Double	S-T, R	--	0.57	0.50	108	1.44	4.2
	PS#4	FS + CMG	Double	S-T	--	0.42				2.8
Mahmood and Ingham [3]	AP8	--	--	DF + SS	37	--	--	--	--	17.1
	WTC6	Vertical NSM CFRP bars	Single	DF + SS	79	--	--	113	--	6.6
	WTC7		Double	DF + SS	153	--	--	313	2.77	3.4
	WTC8	Horizontal NSM CFRP bars	Single	DF + SS	65	--	--	76	--	9.3
	WTC9		Double	SS	67	--	--	81	1.07	4.6
Yang et al. [49]	UMA-1	--	--	JS	--	0.79	0.83	--	--	1.0
	UMA-2	--	--	JS	--	0.87				1.0
	ETA1-1	FS +BTRC	Single	OB	--	1.21	1.25	51	--	3.14
	ETA1-2	FS +BTRC	Single	OB	--	1.29				4.24
	ETA3-1	FS +BTRC	Double	TF	--	1.60	1.57	89	1.76	6.93
	ETA3-2	FS +BTRC	Double	TF	--	1.53				6.47
	UMB-1	--	--	JS	--	0.70	0.65	--	--	1
	UMB-2	--	--	JS	--	0.60				1
	ETB1-1	FS +BTRC	Single	OB	--	1.01	1.05	62	--	3.1
	ETB1-2	FS +BTRC	Single	OB	--	1.08				2.73
	ETB2-1	FS +BTRC	Double	TF	--	1.30	1.25	92	1.50	5.22
	ETB2-2	FS +BTRC	Double	TF	--	1.19				6.91

^#^ Description of failure mode is from original resource; S-SC: stair-stepped cracking; SS: shear slide; DF: diagonal failure; FS: full surface strengthening; TC: toe crushing; S: sliding along mortar joints; S-T: combined sliding along mortar joints and tensile rupture of units; R: rupture of the CMF reinforcement; O: out-of-plane deformation; BTRC: basalt textile-reinforced concrete; IMG: inorganic matrix-grid; BTRM: basalt textile-reinforced mortar; TRM: textile-reinforced mortar; TRC: textile-reinforced concrete; CMG: cement based matrix-coated alkali resistant glass grid system; JS: joint sliding; OB: out-of-plane bending; TF: TRC failure. ^&^ 95.5% = (0.43 − 0.22)/0.22; ** 2.28 = 218.2/95.5; ^$^ increment factor defined as ratio of shear capacity increment due to double-sided strengthening over single-sided strengthening.

## Data Availability

The raw data supporting the conclusions of this article will be made available by the authors on request due to privacy.
